# Upper Respiratory Tract Microbiome of Australian Aboriginal and Torres Strait Islander Children in Ear and Nose Health and Disease

**DOI:** 10.1128/Spectrum.00367-21

**Published:** 2021-10-20

**Authors:** Andrea Coleman, Julian Zaugg, Amanda Wood, Kyra Cottrell, Eva Grahn Håkansson, Jasmyn Adams, Matthew Brown, Anders Cervin, Seweryn Bialasiewicz

**Affiliations:** a The University of Queensland Centre for Clinical Research, Herston, Australia; b Townsville University Hospital, Townsville, Australia; c Australian Centre for Ecogenomics, The University of Queensland, St. Lucia, Australia; d Queensland Health Deadly Ears Program, Brisbane, Australia; e Clinical Microbiology, Umeå University, Umeå, Sweden; f Royal Brisbane and Women’s Hospital, Brisbane, Australia; g Queensland Paediatric Infectious Diseases Laboratory, Queensland Children’s Hospital, South Brisbane, Australia; INTHERES

**Keywords:** 16S rRNA, Aboriginal and Torres Strait Islander, *Corynebacterium*, *Dolosigranulum*, Indigenous, Nose, *Ornithobacterium*, otitis media, ecology, microbiome

## Abstract

The objective of this study was to examine the nasal microbiota in relation to otitis media (OM) status and nose health in Indigenous Australian children. Children 2 to 7 years of age were recruited from two northern Australian (Queensland) communities. Clinical histories were obtained through parent interviews and reviews of the medical records. Nasal cavity swab samples were obtained, and the children’s ears, nose, and throat were examined. DNA was extracted and analyzed by 16S rRNA amplicon next-generation sequencing of the V3/V4 region, in combination with previously generated culture data. A total of 103 children were recruited (mean age, 4.7 years); 17 (16.8%) were healthy, i.e., normal examination results and no history of OM. The nasal microbiota differed significantly in relation to OM status and nose health. Children with historical OM had greater relative abundance of *Moraxella*, compared to healthy children, despite both having healthy ears at the time of swabbing. Children with healthy noses had greater relative abundance of Staphylococcus aureus, compared to those with rhinorrhea. *Dolosigranulum* was correlated with *Corynebacterium* in healthy children. Haemophilus and Streptococcus were correlated across phenotypes. *Ornithobacterium* was absent or was present with low relative abundance in healthy children and clustered around otopathogens. It correlated with *Helcococcus* and *Dichelobacter. Dolosigranulum* and *Corynebacterium* form a synergism that promotes upper respiratory tract (URT)/ear health in Indigenous Australian children. *Ornithobacterium* likely represents “*Candidatus* Ornithobacterium hominis” and in this population is correlated with a novel bacterium that appears to be related to poor URT/ear health.

**IMPORTANCE** Recurring and chronic infections of the ear (OM) are disproportionately prevalent in disadvantaged communities across the globe and, in particular, within Indigenous communities. Despite numerous intervention strategies, OM persists as a major health issue and is the leading cause of preventable hearing loss. In disadvantaged communities, this hearing loss is associated with negative educational and social development outcomes, and consequently, poorer employment prospects and increased contact with the justice system in adulthood. Thus, a better understanding of the microbial ecology is needed in order to identify new targets to treat, as well as to prevent the infections. This study used a powerful combination of 16S rRNA gene sequencing and extended culturomics to show that Dolosigranulum pigrum, a bacterium previously identified as a candidate protective species, may require cocolonization with Corynebacterium pseudodiphtheriticum in order to prevent OM. Additionally, emerging and potentially novel pathogens and bacteria were identified.

## INTRODUCTION

Otitis media (OM), an inflammation/infection of the middle ear, is a common pediatric condition (1). In many Indigenous populations globally, however, there is a disproportionately large OM-associated burden, negatively affecting schooling and employment outcomes (1, 2). Previous microbiological studies related to OM in Indigenous populations were largely limited to the main otopathogens (Streptococcus pneumoniae, Haemophilus influenzae, and Moraxella catarrhalis), using culture-dependent methods, and seldom included healthy Indigenous control children (3). One study used 16S rRNA next-generation sequencing (NGS) to explore the middle ear effusion and nasopharyngeal/adenoid microbiota in relation to OM with effusion (OME) in 11 Aboriginal and/or Torres Strait Islander (referred to as Indigenous Australian) children (4), which confirmed the association of otopathogen-containing genera and OME.

We previously used culturomics and species-specific quantitative PCR (qPCR) to explore the nasal microbiota in relation to ear health and OM in 103 Indigenous Australian children (5). We found that children with historical or current OM/upper respiratory tract (URT) infection had large otopathogen loads and higher rates of detection of rhinovirus (5). In contrast, Corynebacterium pseudodiphtheriticum and Dolosigranulum pigrum were associated with URT/ear health (5). However, culture-based analyses can be insensitive to microbial population structure and fastidious or unculturable organisms, such as the recently described “*Candidatus* Ornithobacterium hominis” (6, 7). To address this limitation, 16S rRNA NGS, supplemented with the existing culturomics data, was used to investigate the broader bacterial microbiome and how it relates to ear and nose health and disease in Indigenous Australian children.

## RESULTS

### Patients and samples.

In total, 103 children were recruited; 2 children refused swabbing, resulting in 101 swabs for analysis. All swabs met quality assurance criteria for endogenous retrovirus 3 (ERV3) testing (8). Raw sample 16S rRNA read depth ranged from 149× to 262,880× (median, 119,693×), with quality, contamination, and nonspecific filtering resulting in the remaining read depth ranging from 0× to 163,794× (median, 66,929×). Fourteen samples were subsequently excluded because they did not meet quality control criteria. The agreement between culturomics and 16S rRNA gene sequencing was 59.2%, with a Cohen’s kappa value of 0.08. The low level of agreement was predominately due to the high sensitivity of detection by 16S rRNA gene sequencing, which detected an average of 14.3 more genera per sample (range, 1 to 73 genera per sample), compared to culture (means of 18.0 and 3.7 genera per sample, respectively).

### Nasal microbiota in relation to ear health.

**FIG 1:**
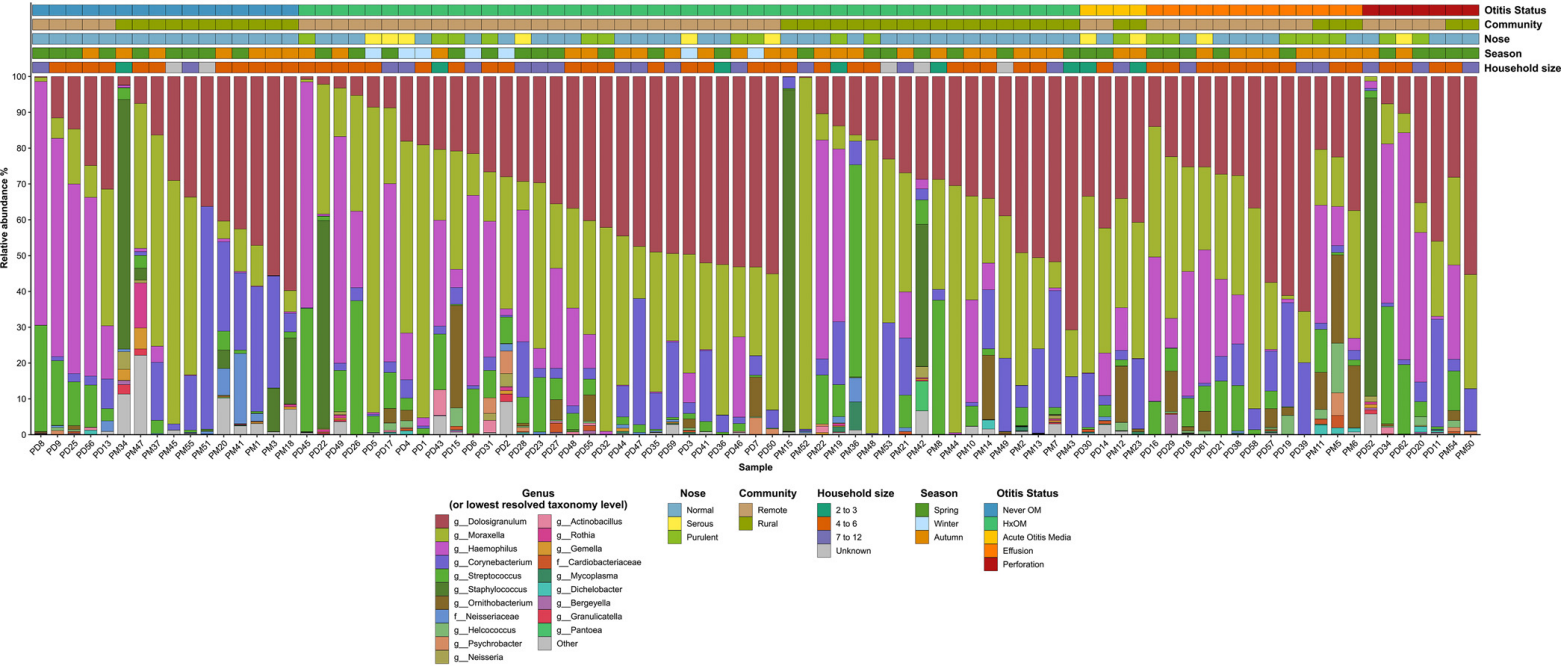
Mean relative microbial abundances of the 20 most abundant genera (or lowest resolved taxonomic level) across all samples, illustrating differences in OM status, community of residence, and other key variables. Microbes with lower abundances have been combined as “other” (gray). To improve interpretability, samples have been ordered by OM status, community, and *Dolosigranulum* abundance.

Only 17 children (16.8%) had no history of OM and normal ear, nose, and throat (ENT) examination results at the time of swabbing (never OM), 7 (6.9%) had a perforated tympanic membrane (TM), 18 (17.8%) had middle ear effusion, 4 (4.0%) had acute OM (AOM), and 55 had a history of OM (HxOM) but a normal TM at the time of swabbing (54.5%) (Table 1). Due to small numbers, AOM samples were excluded from further analyses. There was a significant difference in the nasal microbiota in relation to OM status (permutational multivariate analysis of variance [PERMANOVA], *F* = 2.101, *P = *0.0027), although with dispersion differences (analysis of multivariate homogeneity [PERMDISP], *F* = 3.341, *P = *0.0244). Among children who had healthy TMs at the time of sampling, the HxOM group had greater mean abundance of *Moraxella*, compared to the never-OM group (31.22% versus 20.22%; *P* < 0.05) (Fig. 1; also see Table S1 in the supplemental material). The relative abundance of nine *Dolosigranulum* amplicon sequence variants (ASVs) differed significantly in relation to OM status; ASVs 588 and 2067 were more abundant in children with normal TMs, while ASVs 1030, 1069, and 1528 were more abundant in children with OM (see Table S2) The relative abundance of *Dolosigranulum* was positively correlated with that of *Corynebacterium* in the never-OM group and those of both *Corynebacterium* and *Moraxella* in the HxOM group; there was no significant correlation between *Dolosigranulum* and the other main otopathogen-containing genera (see Fig. S1). Children with effusion had greater mean relative abundance of *Ornithobacterium* (34.1%), compared to the never-OM group (28.4%); although this was nonsignificant according to DESeq2, it was significant according to Dunn’s test (adjusted *P = *0.018; Kruskal-Wallis test, *P = *0.021) (see Fig. S2).

**TABLE 1:** Demographic and clinical details of participants

Characteristic	Data for:		*P* for difference between remote and rural communities
	Remote community (*n* = 59)	Rural community (*n* = 44)	
Female gender (no. [%])	33 (47.7)	21 (47.7)	0.41
Age (mean ± SD) (mo)	57.0 ± 13.4	55.4 ± 18.6	0.61
Educational attendance (no. [%])			<0.001
School	5 (8.5)	14 (31.8)	
Preschool	43 (72.9)	11 (25.0)	
Daycare	5 (8.5)	18 (40.9)	
Home	6 (10.2)	1 (2.3)	
No. of people in the home (mean ± SD)	5.8 ± 2.2	4.9 ± 1.6	0.04
Pneumococcal vaccination (no. [%])^*a*^	56 (94.9)	37 (84.1)	0.04
HxOM group (no. [%])	51 (86.4)	25 (56.8)	<0.001
Never-OM group (no. [%])	5 (8.5)	12 (27.3)	0.01
Historical type of OM (no. [%])			0.07
AOM	25 (42.4)	18 (40.9)	
AOM with perforation	6 (10.2)	2 (8.0)	
OME	3 (5.1)	1 (4.0)	
Chronic suppurative OM	14 (23.7)	1 (4.0)	
Unknown	3 (5.1)	3 (12.0)	
Otoscopic finding at sampling (no. [%])			0.24
Bilateral normal TM	26 (44.1)	29 (65.9)	
Effusion	13 (22.3)	5 (11.4)	
AOM	2 (3.4)	2 (4.5)	
Perforation	5 (8.5)	2 (4.5)	
Unable to visualize TM	13 (22.0)	6 (13.6)	
Nasal discharge at sampling (no. [%])			0.01
None	30 (50.8)	35 (79.5)	
Serous	10 (16.9)	3 (6.8)	
Purulent	19 (32.2)	6 (13.6)	
Oropharynx at sampling (no. [%])			0.73
Tonsillitis	0	0	
Pharyngitis	2 (3.4)	1 (2.3)	
Season of collection (no. [%])			0.01
Winter	7 (11.9)	0	
Spring	29 (49.2)	16 (36.4)	
Summer	0	0	
Autumn	23 (38.9)	28 (63.6)	

a According to the Australian Vaccination Schedule (22).

**FIG 2:**
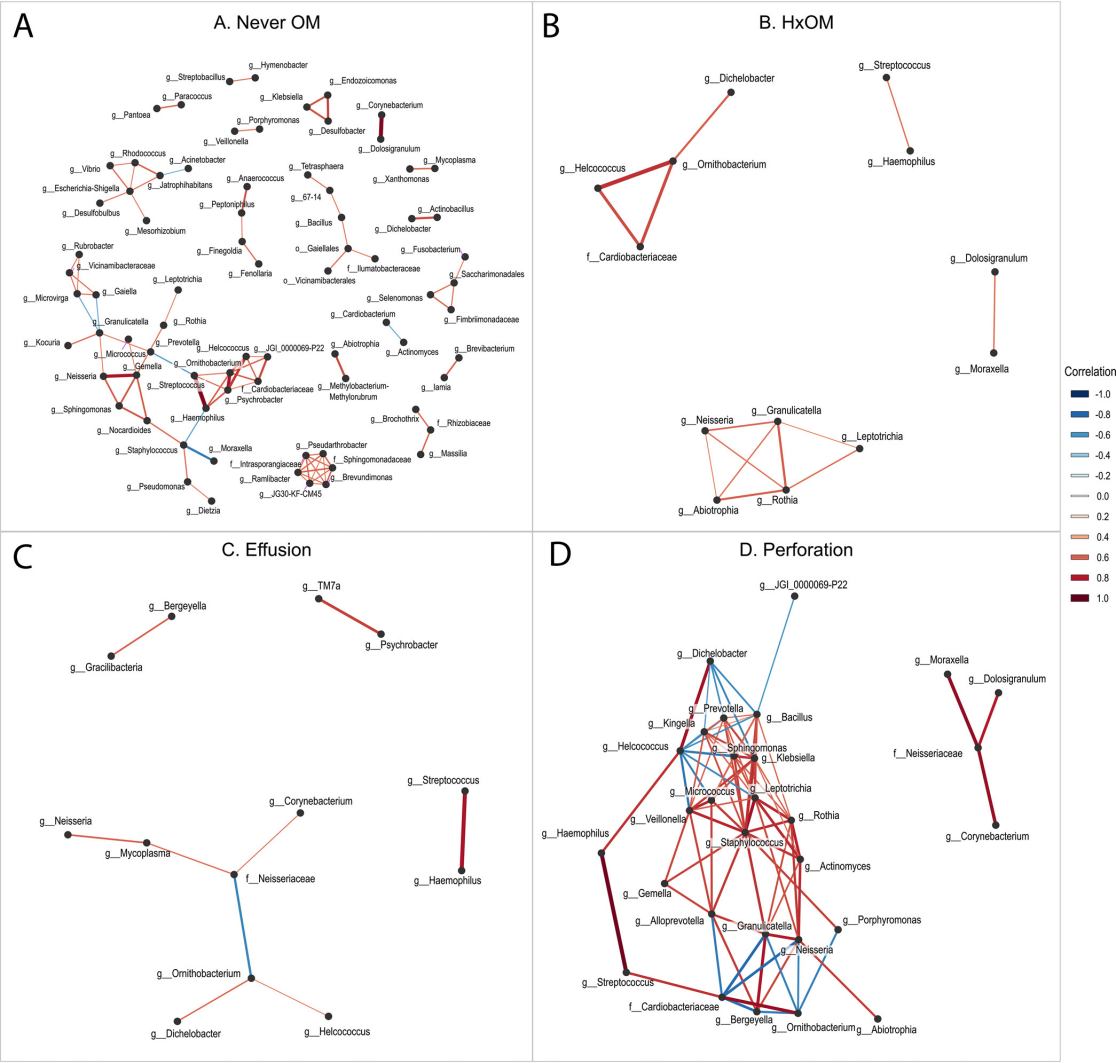
Network correlation analysis, showing differences in relationships of genera in the context of OM status. (A) Never-OM group. (B) HxOM group. (C) Middle ear effusion group. (D) TM perforation group. Connections between genera indicate significant correlation (*P* value of ≤0.05 and absolute correlation of ≥0.5) between their respective abundances. The strength and direction of correlations are indicated by line thickness and color, respectively (e.g., *Dolosigranulum* and *Corynebacterium* show very strong positive correlation in the never-OM group). Genera are labeled by their lowest resolved taxonomic level.

Network analyses showed that taxon correlations largely differed according to OM status, with the notable exception of Streptococcus and Haemophilus, which were correlated across all groups. Never-OM children had a more complex network of correlated genera, compared to the HxOM group, despite both groups having normal TMs at the time of swabbing (Fig. 2). *Dolosigranulum* was positively correlated with different genera across all OM phenotypes, with exception of the effusion group, with *Corynebacterium* in the never-OM group, and with *Moraxella* and *Neisseriaceae* in the HxOM and TM perforation groups, respectively (Fig. 2). Our culturomic data suggested that the species representing the associated genera were Dolosigranulum pigrum and Corynebacterium pseudodiphtheriticum (5). *Ornithobacterium* was correlated with *Helcococcus* and *Dichelobacter* (Fig. 2). There were no significant differences in alpha diversity in relation to OM status (see Table S3).

### Nasal microbiota in relation to nose health.

**FIG 3:**
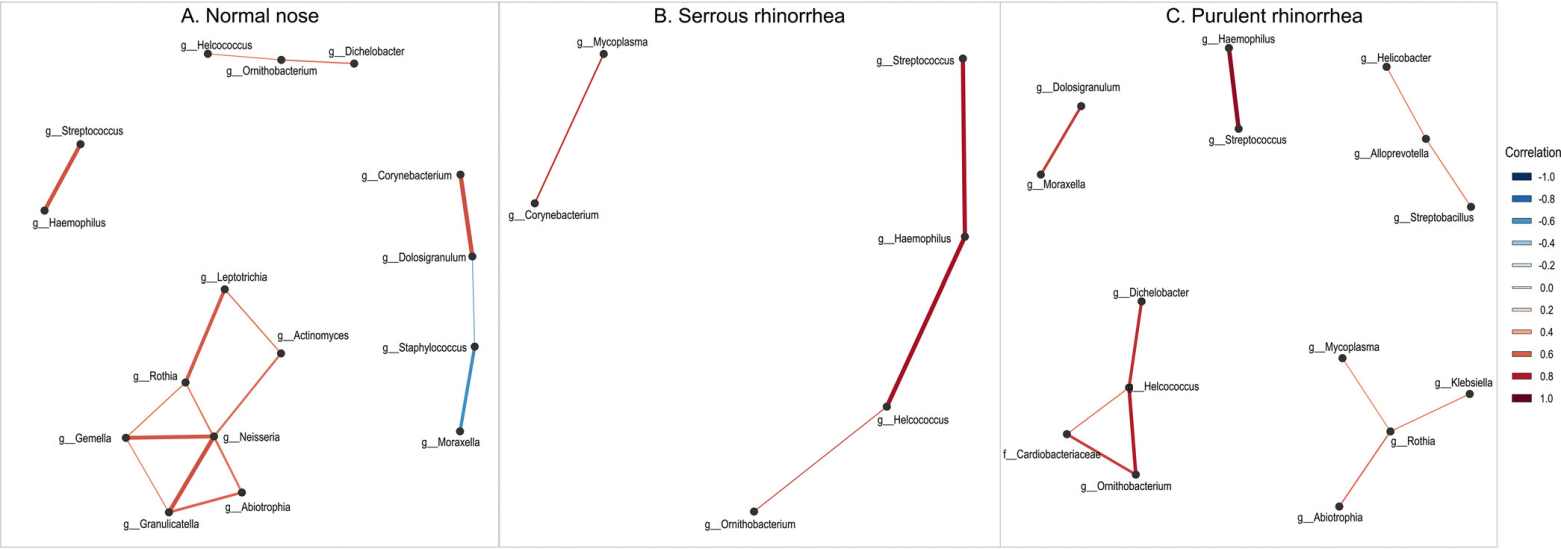
Correlation network analysis of genera in relation to nose health, showing differential *Dolosigranulum* relationships. Connections between genera indicate significant correlation (*P* value of ≤0.05 and absolute correlation of ≥0.5) between their respective abundances. The strength and direction of correlations are indicated by line thickness and color, respectively (e.g., Streptococcus and Haemophilus show very strong positive correlation in all nose status groups but Haemophilus has an additional strong correlation with *Helcococcus* only in the group of children with serous rhinorrhea). Genera are labeled by their lowest resolved taxonomic level.

The nasal microbiota was significantly related to nose health (PERMANOVA, *P < *0.001, *F* = 2.98; PERMDISP, *F* = 2.753, *P = *0.068). Compared to purulent rhinorrhea, children with healthy noses had significantly greater mean relative abundance of Staphylococcus (6.68% versus 0.004%) and *Neisseriacea*e (0.868% versus 0.096%) (all *P* < 0.001) (Fig. 1; also see Table S2). ASV analysis showed that Staphylococcus aureus likely accounted for the Staphylococcus detections. Similar to ear health, multiple *Dolosigranulum* ASVs were detected across nose phenotypes (see Table S2). Network complexity and correlation patterns between *Dolosigranulum* and other bacteria in relation to nose status were similar to those seen within OM status (Fig. 3). Staphylococcus was correlated negatively with *Moraxella* in healthy noses; however, *Ornithobacterium* was present in all phenotypes and was correlated with *Helcococcus*, *Dichelobacter*, and *Cardiobacteriaceae* (Fig. 3). There were no significant differences in alpha diversity in relation to nose health (see Table S3).

### Nasal microbiota in relation to season, household occupancy, and community.

**FIG 4:**
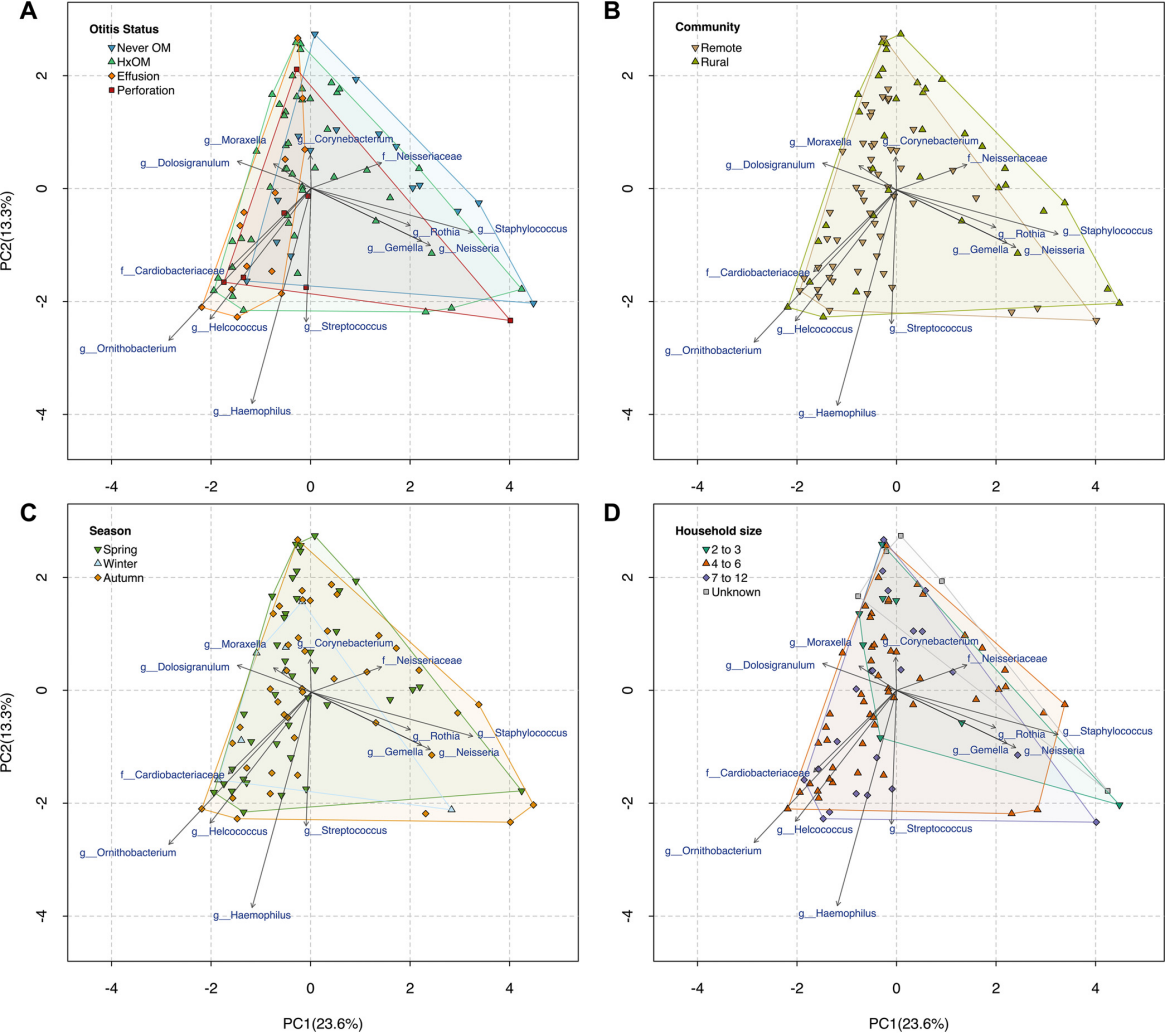
Genus-level PCA, showing the variation in microbial community composition across samples. Groups of samples are indicated by the colored hulls and points, with generally no separation being observed between groups according to OM status (A), community of residence (B), season of swab collection (C), and number of people residing in the household (D). A lack of separation between groups suggests that the microbial communities are largely similar between samples.

No relationship was found between nasal microbiota and season (PERMANOVA, *P = *0.456, *F* = 1.00; PERMDISP, *P = *0.192, *F* = 1.619) or household occupancy (PERMANOVA, *P = *0.748, *F* = 0.791; PERMDISP, *P = *0.844, *F* = 0.181). There were no significant differences in relative abundance or alpha diversity for these variables (Fig. 1; also see Table S3). The nasal microbiota differed significantly in relation to community of residence (PERMANOVA, *P* < 0.001, *F* = 3.71), although with dispersion differences (PERMDISP, *F* = 7.87, *P = *0.005). No separation was observed between the two communities in principal-component analysis (PCA) (Fig. 4).

## DISCUSSION

We demonstrated that the nasal microbiota of Indigenous Australian children was related to ear and nose health. Healthy children with no history of OM showed a relationship between *Dolosigranulum* and *Corynebacterium*. We detected *Ornithobacterium* in children with OM, suggesting a potential role as a novel otopathogen in this population.

In relation to OM status, *Moraxella* had a greater relative abundance in HxOM children, compared to children with no history of OM, despite both groups having healthy ears at the time of swabbing. In children with healthy noses, there was a negative correlation between *Moraxella* and Staphylococcus*. Moraxella* strains are common nasal colonizers whose abundance can increase during acute respiratory infections, leading to prolonged periods of enrichment within the nasal microbiota (9, 10). Thus, the observed increase in *Moraxella* in HxOM children may be a downstream persistent effect of past respiratory infections (e.g., OM), leading to a remodeled microbiome distinct from that of children who did not contract OM. *In vitro* studies demonstrated that some Staphylococcus species can inhibit the growth of M. catarrhalis (11), which may account for their negative correlation within healthy noses in our cohort.

A combination of 16S rRNA NGS and culturomic data strongly suggested that correlations exist between C. pseudodiphtheriticum and D. pigrum in healthy children with no rhinorrhea and no historical OM. In non-Indigenous infants, *Corynebacterium* and *Dolosigranulum* are well recognized as being associated with a stable nasopharyngeal microbiota, conferring URT and ear health (10, 12–15). *In vitro* studies of *Corynebacterium*-*Dolosigranulum* relationships demonstrated complex interactions that were species specific, which may be dependent on the use of host resources (16). However, both C. pseudodiphtheriticum and D. pigrum were required for the inhibition of S. pneumoniae; neither species could inhibit the growth of S. pneumoniae alone (16). These *in vitro* findings corroborate our *in vivo* data and warrant further investigation, particularly with the view toward prevention/control of otopathogen colonization in the nose and consequent ear health benefits.

*Dolosigranulum* was ubiquitous in the nasal microbiota of our population; however, examination at the level of ASVs suggests that this may be a heterogeneous group. The method of ASV analysis has greater precision than analysis of operational taxonomic units (OTUs), which was used in prior URT microbiota research, and thus may be more sensitive in detecting strain-specific differences (17). There is one known species within the *Dolosigranulum* genus; however, our findings suggest the presence of more than one strain or species, particularly given that the 16S rRNA V3/V4 region, as well as the wider D. pigrum genome, has been reported to be highly conserved (16). We did not see an inverse relationship with any of the main otopathogens, as described previously (18), which may be due to this population having a high baseline level of otopathogen colonization (5). There is growing interest in *Dolosigranulum* due to its association with URT and ear health; therefore, the use of whole-genome sequencing and ASV analysis will provide further understanding of the nuances of this nasal commensal.

*Ornithobacterium* was absent or was present with low relative abundance in the nasal microbiota of children with no history of OM. This likely represents “*Candidatus* Ornithobacterium hominis,” a newly described species of *Ornithobacterium* that resides in the nasopharynx and is the only known human species in that genus (7). The role of “*Candidatus* Ornithobacterium hominis” in relation to respiratory/ear disease is still undetermined; however, it was originally found in Australian children and Thai refugee camp infants with high rates of respiratory disease (19, 20). Our findings suggest that *Ornithobacterium* may be associated with poor ear health. Furthermore, the network correlations supported relationships between *Ornithobacterium*, *Helcococcus*, and *Dichelobacter*, which may influence clinical outcomes. Intriguingly, the ASV and correlation network data suggest that there may be novel bacterial species within the nasal microbial ecosystem in genera that currently do not have human representatives (e.g., *Dichelobacter* and *Gracilibacteria*) or have only one species (*Dolosigranulum*). Along with *Ornithobacterium*, these genera warrant further investigations, particularly given their recurring relationships with genera associated with health and disease.

Although this is the largest NGS-based OM study in any Indigenous population to date, limitations exist. Recruitment and sample collection in remote Australian communities are resource- and time-intensive, which affected the sample size. Furthermore, recruitment of healthy children with no history of OM was challenging, despite our community-based sampling, reflecting the large burden of disease in remote Indigenous Australian communities (21). A well-powered, observational, prospective longitudinal birth cohort study would be ideal to elucidate the URT microbial ecology, interactions, and impacts on ear health among Aboriginal and Torres Strait Islander children and, given sufficient resources and time, should be achievable. We found that healthy children with no history of OM appeared to have differences in their nasal microbiota, depending on their community of residence; however, the small sample size limited further subgroup analysis. A well-recognized limitation of 16S rRNA gene sequencing is its poor resolution at the species level. However, a combination of ASV and culturomic data partially overcame this limitation and provided novel species-level insights into the nasal microbial ecology in nose/ear health and disease. It is hoped that, moving forward, methods such as metagenomic shotgun sequencing can be optimized for the URT to provide a more comprehensive assessment of the URT microbiome in relation to health and disease.

In conclusion, our investigation of the nasal microbiota of Indigenous Australian children demonstrated that there is a potential synergism between D. pigrum and C. pseudodiphtheriticum that may be associated with ear and nose health and thus warrants further investigation. Our ASV-level analysis suggested that *Dolosigranulum* is a heterogeneous genus. Finally, we have detected the likely presence of “*Candidatus* Ornithobacterium hominis” and suggestions of other novel species within the nasal microbiota that are associated with poor URT/ear health.

## MATERIALS AND METHODS

Additional details on the methods can be found in the supplemental material.

### Population and sample collection.

Indigenous Australian children 2 to 7 years of age were recruited prospectively from one rural community and one remote community in northern Queensland, Australia, in October 2015 to November 2017. Children who had received antibiotics within 3 weeks before sample collection were excluded (5). The study was approved by the Far North Queensland Human Research Ethics Committee (approval number HREC/15/QCH/10-594).

A detailed description of the cohort, sampling, and clinical data collection was documented previously (5). Briefly, demographic details and ear health history were collected for eligible children from parent interviews and the children’s medical records. Children underwent ENT examinations (including otoscopy). Ear status at the time of swabbing was classified according to the more affected ear. Intranasal mucosal swab (dry FLOQSwabs; Copan Diagnostics, USA) samples used for molecular analysis were collected in parallel with Rayon swab (Transystem Minitip; Copan Diagnostics) samples for culturomics (5). All swab samples were kept at 4°C from the time of collection until arrival at the laboratory 24 to 48 h later. Molecular swab samples were then stored at −80°C.

### DNA extraction and quality assurance.

DNA was extracted via mechanical bead beating and tissue lysis, followed by automated MagNA Pure (Roche Diagnostics, Australia) extraction, as described previously (5). Four clean negative-control swabs were processed in parallel with the sample swabs. The quality of nasal sampling was assessed using a real-time PCR assay targeting the ERV3 marker for human DNA (8). Swab samples that were amplified with cycle thresholds of ≤38 were considered to have adequate nasal epithelial cell content and, by extension, to be of good collection quality. Swab samples yielding cycle thresholds of >38 were excluded from further analysis.

### 16S rRNA gene sequencing.

All sample and negative-control DNA extracts underwent 16S rRNA gene amplification using the 341F/806R primer set, followed by secondary indexing PCR. The equimolar library pool was then sequenced on a MiSeq instrument (Illumina, San Diego, CA, USA) with a 600-cycle v3 kit (2 × 300-bp paired-end reads).

### Sequence data processing.

Primer sequences were removed from demultiplexed reads using Cutadapt (v. 2.6). Reads were filtered and dereplicated using QIIME2 (v. 2019.10.0), and chimeras were removed by DADA2. Taxonomy was assigned to the resulting ASVs by aligning each (classify-consensus-BLAST) against the nonredundant SILVA database (release 138).

### Data analysis and statistics.

Amplicon data analyses were performed in R (v. 4.0.2). ASVs that were not bacterial, fungal, or archaeal in origin, classified at or below the phylum level, or that were classified as chloroplast or mitochondrial were discarded. Putative contaminants were identified using decontam (v. 1.8.0) and microDecon (v. 1.0.2) and removed. ASVs with relative abundance of <0.05% were also removed, and samples with less than 4,000 reads remaining were then discarded. Sample depth was limited to a maximum of 50,000 reads by using the rarefy function in vegan (v. 2.5-6).

Vegan was used to perform PCA, PERMANOVA, and PERMDISP on centered log-ratio-transformed ASV counts collapsed to the genus level. Differentially abundant ASVs and genera were identified using DESeq2 (v. 1.28.1). Alpha diversity metrics Chao1, Shannon, and Simpson were calculated using phyloseq (v. 1.32.0) on samples rarefied to 10,000 reads. Significant differences in alpha diversity distributions were determined with either Mann-Whitney U tests or Kruskal-Wallis and Dunn’s multiple-comparison tests, corrected for multiple testing using the Benjamini-Hochberg method. FastSpar (v. 0.0.10) was used for correlation analysis of genera.

### Culturomic analysis.

Culture-based swab samples were processed using an expanded agar protocol under aerobic and anaerobic conditions, with Vitek matrix-assisted laser desorption ionization–time of flight mass spectrometry (MALDI-TOF MS) (bioMérieux) isolate identification as described previously (5). Agreement between culture and 16S rRNA gene sequencing results was assessed using Cohen’s kappa.

### Data availability.

Amplicon sequencing data have been deposited in the NCBI Sequence Read Archive (SRA) under BioProject number PRJNA684919, with GenBank accession numbers SRR13264782 to SRR13264885.
