# Supplementary material for: Upper Respiratory Tract Microbiome of Australian Aboriginal and Torres Strait Islander Children in Ear and Nose Health and Disease
Source: Microbiol Spectr. 2021 Oct 20;9(2):e00367-21. doi: 10.1128/Spectrum.00367-21 (PMC8528113; doi:10.1128/Spectrum.00367-21)
Supplement: SUPPLEMENTAL FILE 1 — Supplemental material. Download SPECTRUM00367-21_Supp_1_seq5.pdf, PDF file, 0.5 MB [file spectrum00367-21_supp_1_seq5.pdf]

Supplementary material

Supplementary methods

Sequencing

PCR amplicons were generated using the primers 341F (CCTAYGGGRBGCASCAG) and 806R (GGACTACNNGGGTATCTAAT) under the following conditions: 98°C for 30s, and 30 cycles if 98°C for 10s, 60°C for 10s, and 72°C for 30s, with a final extension step of 72°C for 5m. Thermocycling was completed with an Applied Biosystem 384 Veriti and using Platinum SuperFi II mastermix (Life Technologies, Australia) for the primary PCR. The first stage PCR was cleaned using magnetic beads, and samples were visualised on 2% Sybr Egel (Thermo-Fisher). A secondary PCR to index the amplicons was performed with Platinum SuperFi II mastermix (Life Technologies, Australia). The resulting amplicons were cleaned again using magnetic beads, quantified by fluorometry (Promega Quantifluor) and normalised. The eqimolar pool was cleaned a final time using magnetic beads to concentrate the pool and then measured using a High-Sensitivity D1000 Tape on an Agilent 2200 TapeStation. The pool was diluted to 5nM and molarity was confirmed again using a Qubit High Sensitivity dsDNA assay (ThermoFisher). This was followed by sequencing on an Illumina MiSeq (San Diego, CA, USA) with a V3, 600 cycle kit (2 x 300 base pairs paired-end).

Sequence data processing

Primer sequences were removed from forward and reverse de-multiplexed reads using cutadapt (ver. 2.64)(1), with reads not containing primers discarded (--discard-untrimmed). Trimmed reads were processed using QIIME2 (ver. 2019.10.0)(2) for amplicon sequence variant (ASV)(3) selection and taxonomy assignment. Specifically, reads were merged and de-noised (filtered, dereplicated and chimeras identified and removed) using DADA2 (--p-

trunc-len-f 250 and --p-trunc-len-r 230)(4). The taxonomy for each ASV was assigned by aligning each ASV sequence against the combined non-redundant 16S and 18S SILVA database (5) (release 138, clustered at 99% identity) using the classify-consensus-blast function with default parameters.

Data analysis and statistics

Amplicon data analyses were performed in R (ver. 4.0.2). ASVs that were not bacterial, fungal or archaeal in origin, did not receive a classification at the phylum level or below, or that were classified as chloroplast or mitochondria, were discarded.

Putative contaminants were identified using both the decontam (ver. 1.8.0; threshold = 0.5)(6) and microDecon (ver. 1.0.2; runs = 2)(7) packages and removed. Negative samples were discarded after contaminants were removed from all other samples. ASVs with a relative abundance less than 0.05% in all samples were removed, with those samples with less than 4,000 reads remaining then discarded. After removal of low depth samples, 87 samples remained for further analysis. Sample depth ranged from 4,000 to more than 160,000 reads; to minimise issues arising from comparing samples with large differences in read numbers, sample depth was limited to a maximum of 50,000 reads by rarefying using the rrarefy function in the vegan package (ver. 2.5.6)(8). Rarefaction removed reads from 70/87 samples. The number of ASVs removed from these 70 samples ranged between 0-1 (median 0), or proportionally 0.06-69.46% of the reads (median 34.96%).

To account for the compositional nature of the data, ASV counts were transformed to centred log-ratio (clr) values prior to principal-component analysis (PCA), permutational multivariate analysis of variance (PERMANOVA) and analysis of multivariate homogeneity

(PERMDISP; beta dispersion). The rda function from the vegan package was used to perform PCA on clr-transformed ASV counts collapsed to the genus level. PCA figures were created with base R graphics and vegan functions. PERMANOVA and PERMDISP were performed using the adonis and betadisper functions from the vegan package, respectively, with Euclidean distances (also known as the Aitchison distance when counts have been clr transformed) and 999 permutations.

Alpha diversity metrics Chao1 (richness), Shannon (diversity) and Simpson (evenness) were calculated using the phyloseq package (ver. 1.32.0)(9) at the genus level on samples rarefied to 10,000 reads. Significant differences in alpha diversity distributions were determined through Kruskal-Wallis tests followed by Benjamini and Hochberg corrected Dunn's multiple comparisons tests.

Differentially abundant ASVs and genera with ≥50 reads in at least one sample were identified using the Wald test in the DESeq2 package (ver. 1.28.1; fitType = parametric)(10), with *p*-values corrected for multiple testing using the Benjamini and Hochberg method. DESeq2 fits count data to a negative binomial generalised linear model and tests for significant differences between groups. Differential abundance calculations were performed on all samples, and separately on those samples from either remote or rural communities. Result tables were extracted using the results function in DESeq2 with both independent filtering and Cooks cut-off set to false and alpha set to 0.05.

Complementary to DESeq2, differentially abundant genera were also identified by comparing relative abundance distributions of genera with Kruskal-Wallis tests followed by Benjamini and Hochberg corrected Dunn's multiple comparisons tests.

FastSpar (ver. 0.0.10)(11) was used for correlation analysis of genera, with the statistical significance of the correlations calculated using 1,000 bootstrap replicates. Correlation

network figures were limited to significant correlations (*p* value ≤ 0.05 and absolute correlation ≥ 0.5) and were created using the tidygraph (ver. 1.2.0)(12) and ggraph (ver. 2.0.4)(13) packages. Stacked bar graphs and boxplots were created with ggplot2 (ver. 3.3.2)(14). Feature correlation bar graphs were created with base R graphics.

Culturomic analysis and real-time qPCR

Full details of culture-based sample processing and analysis have previously been published (15). In brief, culture-based swabs were processed using an expanded agar protocol under aerobic and anaerobic conditions. Morphological unique isolates were identified using Vitek MS MALDI-TOF (bioMérieux). Isolates that were unable to be identified using MALDI-TOF were than identified by sequencing of the V3-V4 region of the 16S rRNA gene. MALDI-TOF and 16Sr RNA sequenced isolated were combined for culturomic analysis. The extracted nucleic acid from the nasal swabs were tested for *S. pneumoniae*, *H. influenzae*, and *M. catarrhalis* bacterial loads using species-specific qPCRs (15-18).

Variable	Taxonomy	log2FoldChange	*p* adj.
Otitis Status			
Effusion v Never OM	*Gracilibacteria* (JGI_0000069-	19.389	<0.001
	P22)	8.715	0.019
HxOM v Never OM	*Cardiobacteriaceae*	26.423	<0.001
	Gracilibacteria (JGI_0000069-	7.330	0.006
	P22)	2.371	0.031
Never OM v perforation	*Cardiobacteriaceae*	-25.456	<0.001
	Moraxella		
	Gracilibacteria (JGI_0000069-		
	P22)		
Nose			
Healthy v purulent	*Neisseriaceae*	5.759	<0.001
	Staphylococcus	8.821	<0.001
Community			
Remote v rural	*Gracilibacteria*	25.658	<0.001
	Actinobacillus	6.459	<0.001
	Staphylococcus	-4.693	0.027
Season			
Autumn v winter	*Gracilibacteria* (JGI_0000069-	23.665	<0.001
Spring v winter	P22)	22.333	<0.001
	Gracilibacteria (JGI_0000069-		
	P22)		
Household occupants			
2-3 v 4-6	*Helcococcus*	-23.838	<0.001

	Dichelobacter	-21.628	<0.001
	Gracilibacteria (JGI_0000069-	-21.774	<0.001
	P22)	-23.446	<0.001
	Psychrobacter	-8.373	0.028
2-3 v 7-12	*Staphylococcus*	-25.611	<0.001
	Helcococcus	-22.592	<0.001
	Dichelobacter	-22.222	<0.001
	Gracilibacteria (JGI_0000069-	-21.046	<0.001
	P22)		
	Psychrobacter		

Supplementary Table 1: Significantly differentially abundant genera according to DESeq2

Note: adj.= adjusted, OM = otitis media, HxOM = History of OM, but healthy tympanic membrane at time of collection.

Variable	Group_1	Group_2	Taxonomy	ASV_ID	log2FoldChange	*p* adj.
Otitis						
Status	Effusion	HxOM	*Dolosigranulum*	ASV2067	-21.864	<0.001
	Effusion	HxOM	*Dolosigranulum*	ASV588	-20.761	<0.001
	Effusion	HxOM	*Dolosigranulum*	ASV1404	-20.284	<0.001
	Effusion	Never OM	*Dolosigranulum*	ASV1528	25.580	<0.001
	Effusion	Never OM	*Dolosigranulum*	ASV1030	24.353	<0.001
	Effusion	Never OM	*Dolosigranulum*	ASV1476	25.681	<0.001
	Effusion	Never OM	*Dolosigranulum*	ASV1624	25.253	<0.001
	Effusion	Never OM	*Dolosigranulum*	ASV1069	24.600	<0.001
	Effusion	Never OM	*Dolosigranulum*	ASV1404	25.471	<0.001
	Effusion	Never OM	*Dolosigranulum*	ASV2067	-21.734	<0.001
	Effusion	Never OM	*Dolosigranulum*	ASV588	-19.821	0.002
	HxOM	Never OM	*Dolosigranulum*	ASV1404	45.755	<0.001
	HxOM	Never OM	*Dolosigranulum*	ASV1528	25.779	<0.001
	HxOM	Never OM	*Dolosigranulum*	ASV1030	25.248	<0.001
	HxOM	Never OM	*Dolosigranulum*	ASV1476	25.103	<0.001
	HxOM	Never OM	*Dolosigranulum*	ASV1624	23.685	<0.001
	HxOM	Never OM	*Dolosigranulum*	ASV1069	23.622	<0.001
	HxOM	Never OM	*Dolosigranulum*	ASV191	11.915	0.001
	HxOM	Perforation	*Dolosigranulum*	ASV588	38.712	<0.001
	HxOM	Perforation	*Dolosigranulum*	ASV2067	33.442	<0.001
	Never OM	Perforation	*Dolosigranulum*	ASV1404	-45.635	<0.001
	Never OM	Perforation	*Dolosigranulum*	ASV1528	-26.305	<0.001
	Never OM	Perforation	*Dolosigranulum*	ASV588	37.772	<0.001

	Never OM	Perforation	*Dolosigranulum*	ASV2067	33.312	<0.001
	Never OM	Perforation	*Dolosigranulum*	ASV1030	-24.961	<0.001
	Never OM	Perforation	*Dolosigranulum*	ASV1476	-24.835	<0.001
	Never OM	Perforation	*Dolosigranulum*	ASV1624	-26.434	<0.001
	Never OM	Perforation	*Dolosigranulum*	ASV1069	-26.462	<0.001
Nose	Normal	Purulent	*Dolosigranulum*	ASV1624	-25.093	<0.001
	Normal	Purulent	*Dolosigranulum*	ASV588	25.442	<0.001
	Normal	Purulent	*Dolosigranulum*	ASV1069	-23.104	<0.001
	Normal	Serous	*Dolosigranulum*	ASV1624	-24.551	<0.001
	Normal	Serous	*Dolosigranulum*	ASV588	21.882	<0.001
	Purulent	Serous	*Dolosigranulum*	ASV1069	27.700	<0.001

Supplementary Table 2: Significantly differentially abundant Dolosigranulum ASVs in relation to otitis status and nose health.

Note: adj.= ajusted, OM = otitis media, HxOM = History of OM, but health tympanic membrane at time of collection.

Variable	Group_1	Group_2	Shannon Dunn *p* adj.	Shannon KrusW *p* value	Simpson Dunn *p* adj.	Simpson KrusW *p* value	Chao1 Dunn *p* adj.	Chao1 KrusW *p* value
Otitis Status	Effusion	HxOM	0.902	0.544	0.258	0.182	0.552	0.544
	Effusion	Never OM	0.679	0.544	0.190	0.182	0.025	0.544
	Effusion	Perforation	0.994	0.544	0.406	0.182	0.423	0.544
	HxOM	Never OM	1.000	0.544	0.544	0.182	0.041	0.544
	HxOM	Perforation	0.998	0.544	0.894	0.182	0.492	0.544
	Never OM	Perforation	1.000	0.544	0.761	0.182	0.442	0.544
Nose	Normal	Purulent	0.535	0.326	0.405	0.291	0.632	0.326
	Normal	Serous	0.573	0.326	0.699	0.291	0.786	0.326
	Purulent	Serous	0.973	0.326	0.798	0.291	0.905	0.326
Community*	Rural	Remote	0.948		0.621		0.350	
Household occupancy	2 to 3	4 to 6	0.797	0.606	1.000	0.883	0.906	0.606
	2 to 3	7 to 12	0.895	0.606	0.912	0.883	1.000	0.606
	2 to 3	Unknown	0.749	0.606	0.937	0.883	0.994	0.606

	4 to 6	7 to 12	0.736	0.606	1.000	0.883	0.981	0.606
	4 to 6	Unknown	1.000	0.606	1.000	0.883	0.767	0.606
	7 to 12	Unknown	0.734	0.606	1.000	0.883	1.000	0.606
Season	Autumn	Spring	0.560	0.400	1.000	0.898	0.210	0.400
	Autumn	Winter	0.959	0.400	0.978	0.898	0.257	0.400
	Spring	Winter	0.775	0.400	1.000	0.898	0.690	0.400

Supplementary Table 3: Differences in alpha diversity according to otitis status, nose health, community, household occupancy, and season of collection.

Note: * Mann-Whitnney U. adj.= adjusted, OM = otitis media, HxOM = History of OM, but healthy tympanic membrane at time of collection.

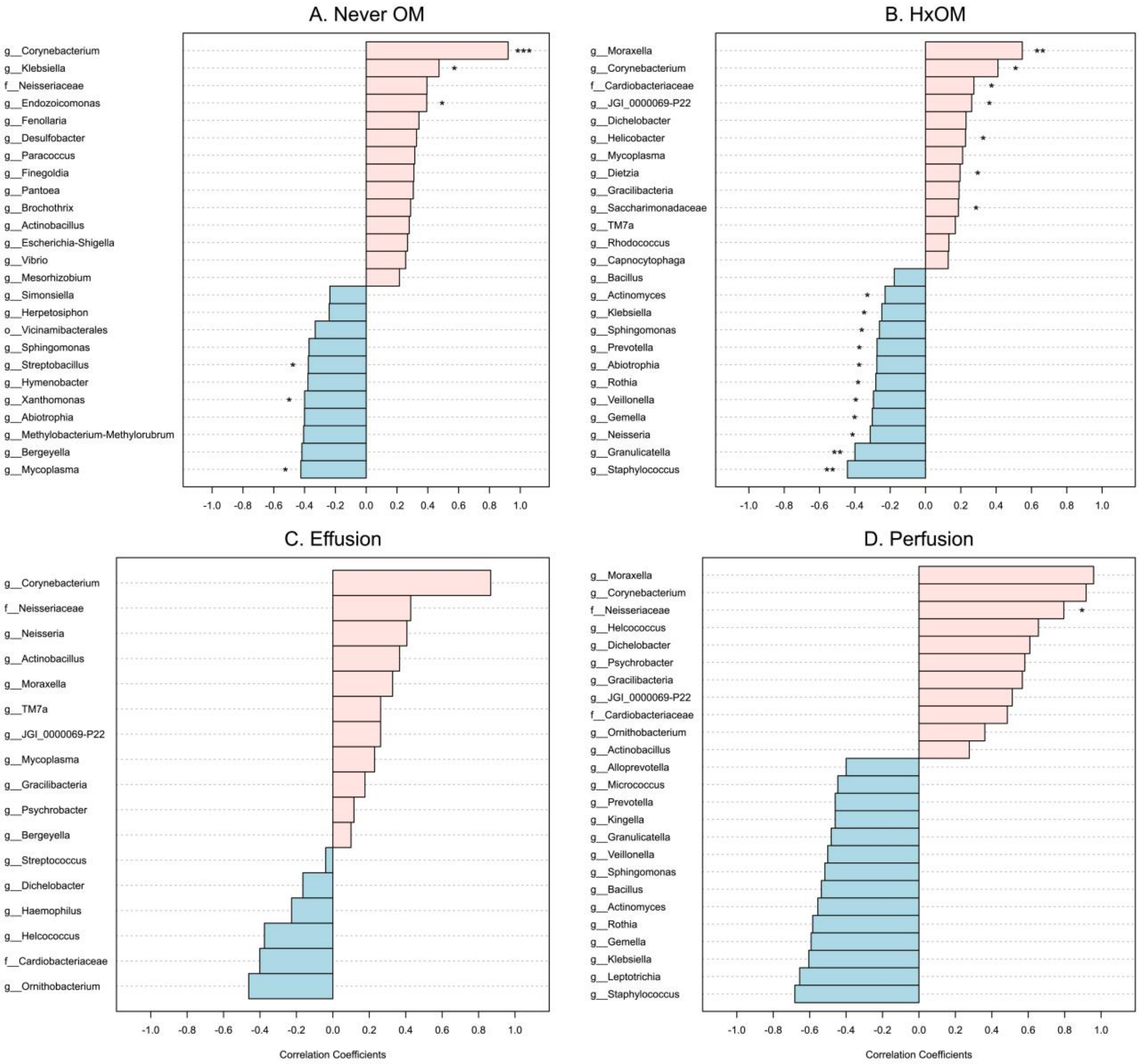

Supplementary Figure 1: Dolosigranulum significantly correlated with Corynbacterium in Never OM and Moraxella in HxOM. Correlations between the relative abundance of Dolosigranulum and the top 25 genera in each group according to FastSpar network analysis. NB: not all groups had 25 genera.

Note: * = p ≤ 0.05, ** = p ≤ 0.01 and *** = p ≤ 0.001. OM = otitis media, HxOM = History of OM, but health tympanic membrane at time of collection.

A)

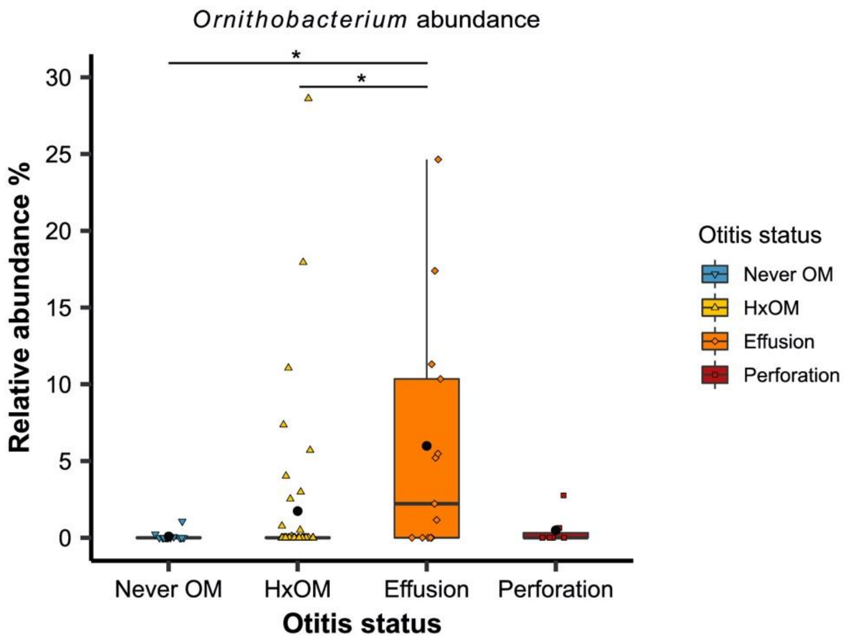

B)

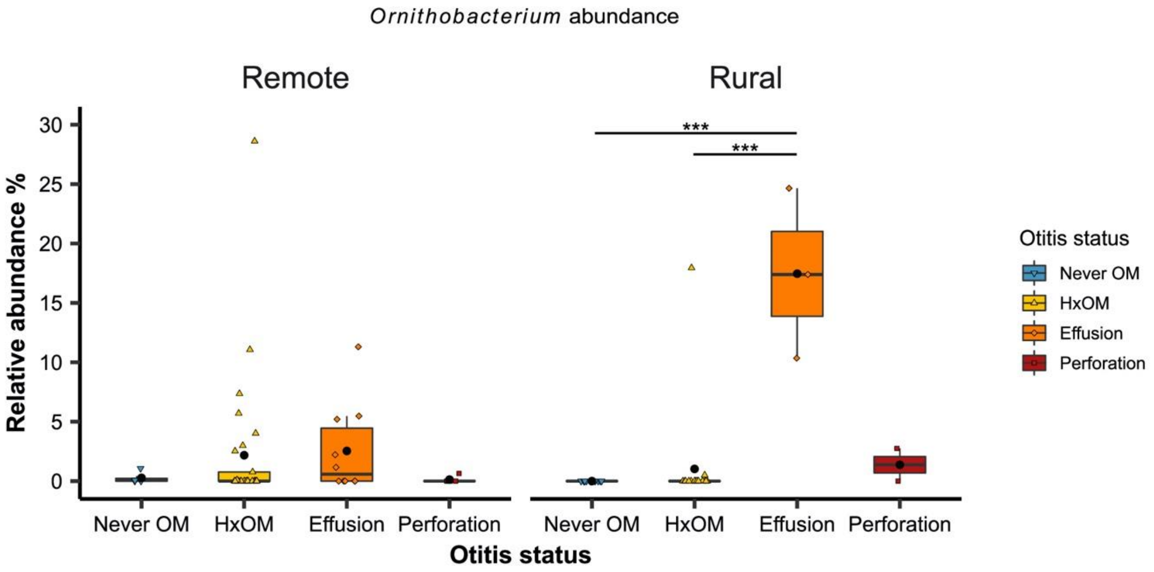

Supplementary Figure 2: Children with effusion had higher mean relative abundance of Ornithobacterium, compared to never OM. Tukey style box plots showing the relative abundances (normalised 16S rRNA read counts) of the Ornithobacterium genus within nasal samples. A) Ornithobacterium relative abundance in relation to otitis status; B) Ornithobacterium relative abundance in relation to otitis statis and community of residence. Bars indicate median +/- 1.5 × interquartile range and the mean relative abundance is indicated by the black dot. Significant differences between groups, calculated by Dunn's multiple comparisons tests, are indicated by * = $p \leq 0.05$, ** = $p \leq 0.01$ and *** = $p \leq 0.001$.

References:

1. Martin M. Cutadapt removes adapter sequences from high-throughput sequencing reads. EMBnet J. 2011;17:10-2.

2. Bolyen E, Rideout JR, Dillon MR, Bokulich NA, Abnet CC, Al-Ghalith GA, et al. Reproducible, interactive, scalable and extensible microbiome data science using QIIME 2. Nature Biotechnology. 2019;37(8):852-7.

3. Callahan B, McMurdie P, Holmes S. Exact sequence variants should replace operational taxonomic units in marker-gene data analysis. ISME J 2017;11:2639-43.

4. Callahan B, McMurdie P, Rosen M, Han A, Johnson A, Holmes S. DADA2: High-resolution sample inference from Illumina amplicon data. Nat Methods 2016;13:581-3.

5. Quast C, Pruesse E, Yilmaz P, Gerken J, Schweer T, Yarza P, et al. The SILVA ribosomal RNA gene database project: improved data processing and web-based tools. Nucleic Acids Res. 2013;41(Database issue):D590-D6.

6. Davis N, Proctor D, Holmes S, Relman D, Callahan B. Simple statistical identification and removal of contaminant sequences in marker-gene and metagenomics data. Microbiome. 2018;6:266.

7. McKnight D, Huerlimann R, Bower D, Schwarzkopf L, Alford R, Zenger K. microDecon: A highly accurate read-subtraction tool for the post-sequencing removal of contamination in metabarcoding studies. Environmental DNA. 2019;1:14-25.

8. Oksanen J, Blanchet F, Kindt R, Legendre P, O’Hara R, Simpson G, et al. Package ‘vegan’: Community Ecology Package. 1.17-2 ed2010.

9. McMurdie P, Holmes S. phyloseq: an R package for reproducible interactive analysis and graphics of microbiome census data. PloS ONE. 2013;8:e61217.

10. Love M, Huber W, Anders S. Moderated estimation of fold change and dispersion for RNA-seq data with DESeq2. Genome biology. 2014;15:550.

11. Watts S, Ritchie S, Inouye M, Holt K. FastSpar: rapid and scalable correlation estimation for compositional data. Bioinformatics. 2019;36:1064-6.

12. Pedersen T. tidygraph: A Tidy API for Graph Manipulation. 2018.

13. Pedersen T. ggraph: An Implementation of Grammar of Graphics for Graphs and Networks. 2018.

14. Wickham H. ggplot2: elegant graphics for data analysis. Springer; 2016.

15. Coleman A, Bialasiewicz S, Marsh R, Grahn Håkansson E, Cottrell K, Wood A, et al. Upper respiratory microbiota in relation to ear and nose health among Australian Aboriginal and Torres Strait Islander children. J Pediatric Infect Dis Soc. 2020;In press.

16. McAvin J, Reilly P, Roudabush R, Barnes W, Salmen A, Jackson G, et al. Sensitive and specific method for rapid identification of *Streptococcus pneumoniae* using Real-Time Fluorescence PCR. J Clin Microbiol. 2001;39(10):3446-51.

17. Greiner O, Day P, Altwegg M, Nadal D. Quantitative detection of *Moraxella catarrhalis* in nasopharyngeal secretion by real-time PCR. Clin Microbiol. 2003;41(4):1386-90.

18. Abdeldaim G, Strålin K, Kirsebom L, Olcén P, Blomberg J, Herrmann B. Detection of Haemophilus influenzae in respiratory secretions from pneumonia patients by quantitative real-time polymerase chain reaction. Diagn Microbiol Infect Dis. 2009;64(4):366-73.
